# Preclinical Evaluation of Fingolimod in Rodent Models of Stroke With Age or Atherosclerosis as Comorbidities

**DOI:** 10.3389/fphar.2022.920449

**Published:** 2022-07-13

**Authors:** Andrea C. Diaz Diaz, Kyle Malone, Jennifer A. Shearer, Anne C. Moore, Christian Waeber

**Affiliations:** ^1^ School of Pharmacy, University College Cork, Cork, Ireland; ^2^ Department of Pharmacology and Therapeutics, University College Cork, Cork, Ireland; ^3^ Department of Pharmacology, University College Cork, Cork, Ireland

**Keywords:** ischemic stroke, immunosuppression, comorbidities, sphingosine 1-phosphate, FTY720

## Abstract

Preclinical data indicate that fingolimod improves outcome post-ischaemia. This study used a rigorous study design in normal male C57BL/6JOlaHsd mice and in mice with common stroke comorbidities to further evaluate the translational potential of fingolimod. Stroke was induced via middle cerebral artery electrocoagulation in 8–9-week old mice (young mice), 18 month old mice (aged mice), and in high-fat diet-fed 22-week old ApoE−/− mice (hyperlipidaemic mice). Recovery was evaluated using motor behavioural tests 3 and 7 days after stroke. Tissue damage was evaluated at 7 days. A lower dose of fingolimod, 0.5 mg/kg, but not 1 mg/kg, increased lesion size but decreased ipsilateral brain atrophy in younger mice, without an effect on behavioural outcomes. Fingolimod-treated aged mice showed a significant improvement over saline-treated mice in the foot fault test at 7 days. Fingolimod-treated hyperlipidaemic mice showed a decreased infarct size but no difference in behavioural performance. Increasing fingolimod treatment time to 10 days showed no benefit in young mice. Pooled data showed that fingolimod improved performance in the foot fault test. Flow cytometry studies showed that fingolimod had marked effects on T cell frequencies in various tissues. The results show that the effects of fingolimod in stroke are less robust than the existing literature might indicate and may depend on the inflammatory status of the animals.

## 1 Introduction

Fingolimod (FTY720) first showed clinical potential after the discovery that it prolonged experimental allotransplant survival, and demonstrated efficacy in animal models of organ transplantation (Brinkmann et al., 2010). It was also shown to prevent ischemia-reperfusion injury in liver (Mizuta et al., 1999) and kidney (Awad et al., 2006). However, clinical trial results showed insufficient benefits in renal transplantation, and development for this indication was halted (Brinkmann et al., 2010). Based on the hypothesis that the fingolimod-mediated reduction of peripheral blood lymphocyte counts and sequestration of lymphocytes into lymph nodes might be beneficial in the treatment of autoimmune disorders, clinical development was reoriented to multiple sclerosis, for which this drug (Gilenya) is now approved.

Fingolimod is an orally active prodrug, phosphorylated into its active metabolite by sphingosine kinase 2. Once activated, fingolimod shows a high affinity for 4 of the 5 known sphingosine 1-phosphate receptors (S1P1, S1P3, S1P4, S1P5) (Brinkmann et al., 2010). S1P receptors are present in all organs and tissues, and they play important roles in cell death and proliferation, vascular functions, as well as in the immune, nervous, and reproductive systems. Fingolimod is an S1P receptor agonist that behaves as a functional antagonist, rapidly downregulating S1P1 receptors expressed by lymphocytes, preventing them from sensing the S1P concentration gradient between blood and secondary lymphoid organs, thereby preventing them from leaving these structures (Brinkmann et al., 2010). Although the ensuing immunosuppression is the most generally accepted mechanism of fingolimod’s efficacy in multiple sclerosis (Jeffery et al., 2016), a direct effect on brain parenchymal cells (Chun and Hartung, 2010) and vasculature (Foster et al., 2008) may also contribute.

While stroke and multiple sclerosis have a different aetiology, some pathophysiological processes play a role in both conditions (Pinheiro et al., 2016; Rommer and Sellner, 2019), suggesting that they may respond to the same pharmacological interventions. Based on this observation, and on the general protective role of S1P receptor stimulation in most cell types (Maceyka et al., 2002), we and others investigated the effect of fingolimod in various rodent models of ischemic [see (Dang et al., 2020) for a recent meta-analysis] and haemorrhagic stroke (Rolland et al., 2011, 2013; Lu et al., 2014; Diaz Diaz et al., 2020). Fingolimod was also tested in three small open-label studies in patients with ischemic stroke, alone (Fu et al., 2014) or in combination with rtPA (Zhu et al., 2015; Tian et al., 2018). Despite the large number of animal studies and the favourable outcome in limited clinical studies, fingolimod has not been studied in large, well-powered experimental ischemic stroke studies, in animal models relevant to the clinical features of stroke patients (older age and comorbidities such as atherosclerosis), and with clinically relevant experimental outcome measures (late time point, and behavioural outcome). The aim of our studies was to fill this gap to confirm the efficacy of fingolimod in an experimental stroke and inform future large-scale clinical trials. We chose a model of permanent cortical ischemia, electrocoagulation of a distal branch of the middle cerebral artery (MCA) because of its ease of implementation and considering that early reperfusion [either spontaneous (Bowler et al., 1998) or following therapeutic intervention (Gauberti et al., 2021)] only occurs in a minority of stroke patients. To test the hypothesis that histological and behavioural outcomes were associated with immunosuppression due to inhibition of lymphocyte egress from lymph nodes, the most likely proximal mechanism of action, we also assessed the proportions of various lymphocyte populations in various tissues.

## 2 Materials and Methods

### 2.1 Experimental Design and Animals

This study was approved (AE19130/P042) by the Irish Health Products Regulatory Authority of Ireland and the Animal Experimentation Ethics Committee at University College Cork. It was performed following guidelines of the National Institute of Health Guide for Care and Use of Laboratory Animals (National Research Council, 2011) and is reported using the ARRIVE guidelines (Kilkenny et al., 2010). An *a priori* sample size calculation was performed to detect a 30% difference in lesion size (based on the overall fingolimod effect size reported in the meta-analysis available when these studies were initiated (Liu et al., 2012)), using variability of the model from preliminary studies. This resulted in *n* = 16 animals per group (with a power of 80%, an alpha of 0.05 and no correction for multiple testing). Exclusion criteria were established in advance; mice that had a severe uncontrollable haemorrhage, thermal or physical damage of the cortical surface at the time of surgery were excluded from the study. Another part of this study, focused more specifically on regulatory T cells (Tregs) and other lymphocyte populations, was published separately (Malone et al., 2021).

Male C57BL/6JOlaHsd mice (7–18 weeks) were from Envigo United Kingdom, aged male C57BL/6NCrl mice from Charles River United Kingdom and male Apoetm1Unc (ApoE^−/−^) mice from Charles River Italy. All mice were allowed to acclimate for at least 10 days before procedures were carried out. Mice were group-housed, 3–4 animals per individually ventilated cages, lined with aspen wood chip; ALPHA-Dri^®^ bedding (Shepherd Specialty Papers, United Kingdom) was used for ApoE^−/−^ mice to help contain excess grease. The facility had a 12 h light/dark cycle and mice were provided additional enrichment and free access to food (2,918 irradiated Teklad global 18% protein rodent diet, Envigo) and water. Mice were regularly monitored and separated in case of aggression.

Drug and vehicle solutions were prepared and their identity was concealed by a researcher not directly involved in the studies. Due to fingolimod’s low solubility, the solution was titrated to pH 7.0. Treatment allocation was randomized upon receipt of the mice using a pseudorandom number generator (randomizer.org). Unblinding only occurred once the data analysis was completed.

### 2.2 Permanent Ischaemia

The stroke surgery was performed as previously described (Llovera et al., 2014). Briefly, mice were anaesthetised with isoflurane in 30% O_2_:70% N_2_. After induction with 3% isoflurane, anaesthesia was maintained with 1.5%–2% isoflurane using a face mask. Rectal temperature was maintained with a rectal probe and homeothermic blanket (NeosBiotec, Spain); the device was set at 37°C and the average recorded body temperature was 36.4°C (35.2–37.4 *n* = 98). An incision was made midway between the left eye and left ear. The parietal muscle was cut away from the insertion point on the skull and retracted using a suture to expose the parietal bone. The bone was thinned using a microdrill, lifted and removed with forceps to allow access to the MCA. In cases where mice did not have a bifurcation of the MCA, mice were euthanised. The dura was pierced and pulled away from the artery. The MCA branches and accessory arteries were occluded by bipolar electrocoagulation with a Bovie Bantam Pro electrosurgical generator and 3 1/2″ McPherson straight forceps (Symmetry Surgical Inc., United States). Once the occlusion was confirmed by nicking the MCA, the bone defect was covered with bone wax and the incision was closed. Mice recovered in a heated chamber at 32°C for 30 min before being returned to their cage. Mice were monitored and their body weight recorded daily.

### 2.3 Behaviour Testing

The cylinder and foot fault tests were selected for the evaluation of symmetry scores associated with unilateral damage to the cortex (Schaar et al., 2010). The tests were performed before surgery and at days 3 and 7 after stroke. Mice in the treatment duration study were evaluated at baseline, 2, 5 and 10 days after surgery with the foot fault test.

The cylinder test was performed in a glass cylinder (12.5 cm × 23.5 cm, DxH). Videos were recorded from above to allow visualisation of the times when mice reared and touched the cylinder wall for as long as it took to observe 20 events. The number of independent touches of the cylinder wall with ipsilateral (Left), contralateral (Right) or both (B) forepaws were counted, and a score was calculated (R-L)/(R + L + B) (Balkaya et al., 2012).

The foot fault, or grid walking, test has been reported to have a high sensitivity to detect deficits in mice with stroke (Schaar et al., 2010). Mice were placed on a 25 cm × 35 cm wire grid with 1 cm^2^ openings and encouraged to walk from one end to the other by placing a cardboard cylinder on the opposite end. Videos were recorded from below the grid to allow visualisation of the paws missing the wire and the total number of steps taken. The first 100 steps were counted along with the number of missed contralateral and ipsilateral steps; the score was calculated as the ratio of contra/ipsi (R/L).

### 2.4 Tissue Processing and Injury Quantification

Mice were euthanized with pentobarbital [20–30 µl i.p.; Euthatal (200 mg/ml)]. Blood was collected from the abdominal aorta and the mice were transcardially perfused with 20 ml of cold PBS (P4417, Sigma-Aldrich). The brain was frozen in 2-methylbutane at −40°C and coated with embedding matrix (M-1, Thermo Fisher). A series of 20 µm-thick sections were cut 500 µm apart using a Leica CM 1900 cryostat. Injury and hemispheric volumes were quantified on images of Haematoxylin/Eosin and NeuN stained sections scanned at 3,200 dpi with an Epson V600 scanner. The lesion and hemisphere boundaries were outlined by an investigator blinded to the treatment groups; specifically, the injury boundary was determined using a microscope at ×10 magnification and the lesion volume was then quantified in the scanned image of each section using ImageJ (v1.51f, NIH). Hemispheric volumes were used for the indirect quantification of tissue loss and/or edema (Sommer, 2016).

#### 2.4.1 Haematoxylin and Eosin

Brain sections were air-dried overnight, fixed in 10% formalin for 5 min and rehydrated in graded alcohols (100%, 95%, and 70%, 2 min each), followed by 2 min in distilled water. Slides were placed in Mayer’s haematoxylin solution (Sigma-Aldrich) for 4 min, rinsed in tap water until the water ran clear and transferred into 0.25% Eosin Y solution for 1 min. Slides were dehydrated through graded alcohols (2 min each), cleared in Histochoice and coverslipped with Permount (Fisher Scientific).

#### 2.4.2 Immunohistochemistry: NeuN Staining

Sections were air-dried overnight, followed by 20 min of fixation in methanol (Sigma-Aldrich) at −20°C. Endogenous peroxidase activity was quenched in cold 10% hydrogen peroxide-methanol solution for 10 min. Sections were blocked with 5% normal goat serum (s-1000, Vector Laboratories) in 0.3% TritonX TBS, incubated with rabbit Anti-NeuN antibody (1:1,500; Abcam ab177487) for 1 h at RT, and with Goat Anti-Rabbit biotinylated IgG (1:250; Abcam ab207995) for 1 h at RT. Slides were washed 3 times with TBS, incubated in avidin-biotin horseradish peroxidase solution (ABC Kit pk-4000, Vector Laboratories) for 30 min and the reaction product visualised with diaminobenzidine (DAB Kit sk-4100, Vector Laboratories). The sections were dehydrated in a series of alcohols, cleared with Histochoice and coverslipped with Permount.

### 2.5 Flow Cytometry

Abdominal aorta blood was collected into EDTA-coated tubes at the experimental endpoint. Following cold PBS perfusion, cervical lymph nodes (LNC), inguinal lymph nodes (LNI), and spleen were harvested into sterile Dulbecco’s PBS (Sigma-Aldrich). Using the plunger of a 3 ml syringe, these tissues were mechanically dissociated in approximately 3 ml PBS in a sterile 6-well plate. The resulting cell suspensions were passed through a 70 μm cell strainer and collected in a 50 ml conical tube. Spleen and blood samples were resuspended in 5 ml of 1X RBC Lysis Buffer (eBioscience) and incubated for 5 min at RT. The lysis reaction was stopped by adding 20 ml of 1X PBS. Cells were washed twice with 1X PBS, resuspended in PBS, and counted using trypan blue.

Samples were incubated for 5 min with 50 µl of anti-mouse CD16/CD32 (Clone 93, 1:100; eBioscience). The cell suspensions were then stained for anti-mouse CD45 (PerCP-CY5.5) (30-F11, 1:100), CD3 (PE-Cy7) (145-2C11, 1:100), CD4 (FITC) (RM4-5, 1:800), CD8 (Pacific Blue) (5H10, 1:100), and CD25 (APC) (PC61.5, 1:100) (eBioscience). A live/dead stain (1:10,000 solution) was also added to each sample (Fixable Viability Dye eFluor 780, eBioscience). The samples were incubated in the dark for 30 min at 2°C–8°C, washed, fixed, and resuspended in PBS. Flow cytometric analysis was performed with an LSRII flow cytometer (Becton Dickinson). Compensation control was set using BD CompBead Anti-Rat/Anti-Hamster Particles. Data were analysed using FlowJo (v10). Gates were set according to unstained samples and fluorescent minus one (FMO) controls (see Supplementary Figure S1 for gating strategy). Absolute cell counts for all tissues were calculated based on the instructions provided with the CountBright Absolute Counting Beads (Molecular Probes).

### 2.6 Study Design

#### 2.6.1 Dose Response

**FIGURE 1:**
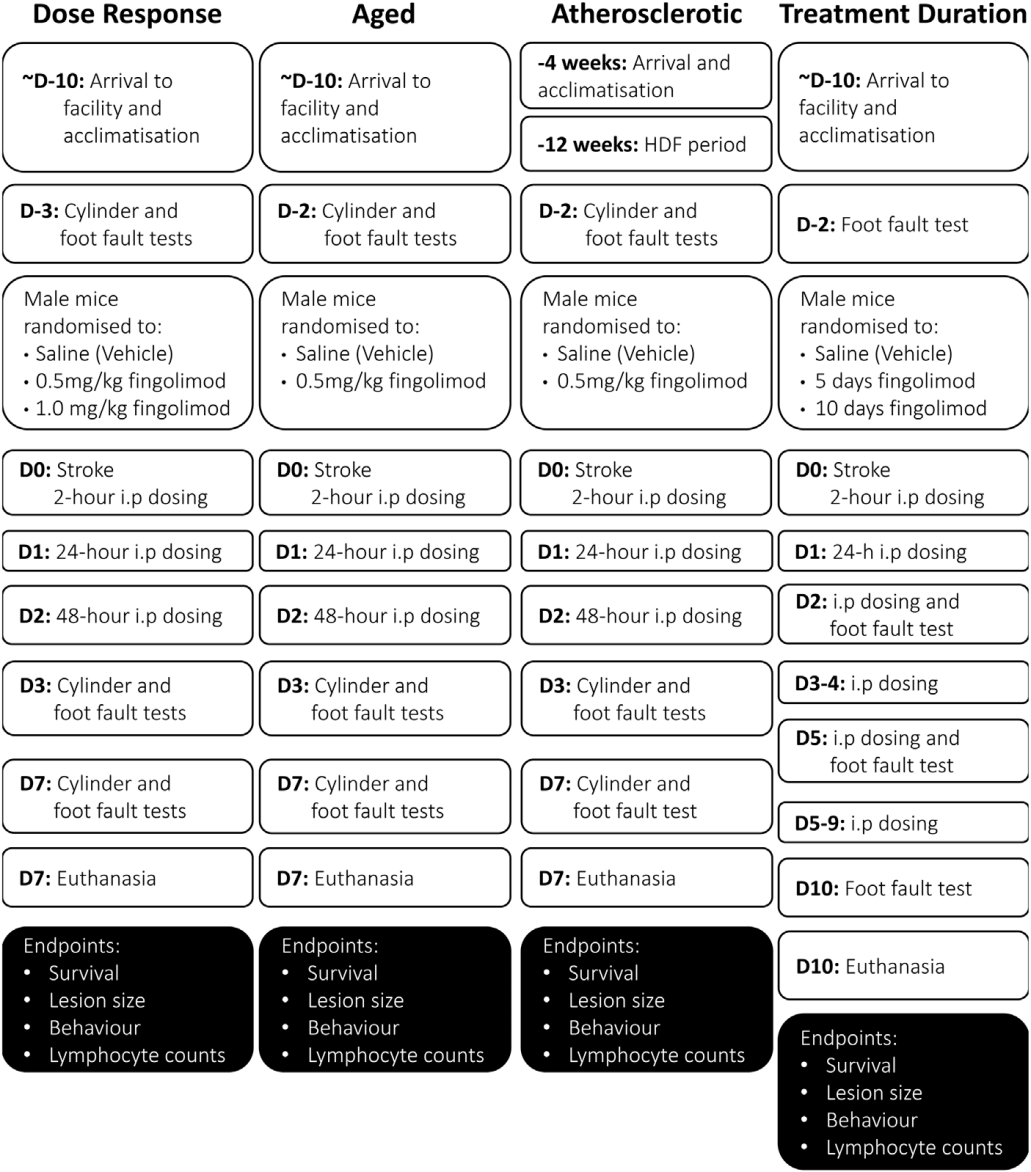
Flowcharts illustrating the experimental design and timeline in the various sub-studies included in these studies (D, day; W, week). Note that the acclimatization period was at least 10-days for all mice, and may have been as much as 2-weeks long for some mice.

Forty nine male C57BL/6JOlaHsd mice (15–17 weeks of age) (Envigo, United Kingdom) were used to determine an optimal dose of fingolimod. Mice received intraperitoneal injections of vehicle (saline), 0.5 mg/kg, or 1.0 mg/kg fingolimod (Liu et al., 2012; Dang et al., 2020), 2, 24 and 48 h after MCA occlusion. This treatment regimen was based on previous studies by our group. (Wei et al., 2010; Campos et al., 2013). Once-daily dosing regimen was chosen based on the extended half-life of fingolimod (Snelder et al., 2014). Behavioural tests were performed at baseline, and 3 and 7 days following stroke (cylinder and foot fault) (Figure 1).

#### 2.6.2 Aged Mice

Thirty two C57BL/6NCrl mice (Charles River), 71–73 weeks of age, were used for this study. Although senescence is observed in mice at about 18 months (78 weeks), we chose a relatively younger age [the equivalent of 56 human years (Dutta and Sengupta, 2016)] due to the high mortality of these mice following experimental stroke. Mice received saline or fingolimod (0.5 mg/kg) intraperitoneally at 2, 24 and 48 h post-MCAO. Behavioural assessments (cylinder and foot fault) were completed at baseline, 3 and 7 days post-stroke.

#### 2.6.3 Atherosclerotic Mice

Hyperlipidaemia and atherosclerosis were modelled with ApoE^−/−^ mice fed a high-fat diet (HFD). Forty ApoE/J (B6.129P2-Apoetm1Unc/J) mice 4–5 weeks of age (Charles River, Italy) were used for this study. Starting at 8 weeks of age, mice were fed an adjusted calorie diet (TD.88137, Envigo, United States) for 12 weeks, during which 2 mice were euthanized. All HFD-fed ApoE^−/−^ mice showed a high aortic plaque burden and elevated serum cholesterol levels (Supplementary Figure S2). Towards the end of the 12 weeks, mice started to present with skin lesions (dermatitis), possibly resulting from excess cholesterol levels together with play-fighting (Zhang et al., 2015). These mice were separated and treated with Calamine lotion after which the dermatitis cleared. Ischaemic stroke was induced after 12 weeks of HFD. Mice were randomized into saline or fingolimod (0.5 mg/kg) groups administered at 2, 24 and 48 h post-MCAO. After the stroke was induced mice were switched back to standard chow (Teklad global 18% protein, Irradiated). Behavioural assessment (cylinder and foot fault) was completed at baseline, 3 and 7 days post-stroke. The atherosclerotic plaque burden was assessed in Oil Red O-stained aortic arch whole-mounts, while blood cholesterol levels were measured with a Cholesterol Fluorometric Assay kit (Cayman Chemical No. 10007640) (see Supplementary Methods and Supplementary Figure S2).

#### 2.6.4 Treatment Duration

Fifty one C57BL/6JOlaHsd mice (Envigo, United Kingdom) at 8–9 and 17–19 weeks of age were used. We evaluated 3 administration regimens: 10 days of saline, 5 days of fingolimod (0.5 mg/kg) followed by 5 days of saline, and 10 days of fingolimod (0.5 mg/kg). The foot fault test was performed at baseline, and on days 2, 5 and 10 post-stroke. The cylinder test was not used for these mice because it seemed less sensitive to treatment effects in the other sub-studies (in which the saline groups consistently showed a score close to 0, suggesting that this test is not suitable to detect motor deficits in our stroke model).

### 2.7 Statistical Analysis

Data were evaluated for normality with a Shapiro-Wilk test. Survival was assessed by Log Rank analysis of the Kaplan-Meier curve. T-tests, one- and two-way ANOVA were performed, as indicated depending on variable and distribution types, with Prism 9 (GraphPad Software version), with significance set at *p* < 0.05. Correlations between experimental outcomes and lymphocyte subpopulation frequencies were investigated using Pearson’s correlation analysis. The heat map of linear correlation was visualized with GraphPad Prism.

## 3 Results

### 3.1 Dose Response

The final group sizes were *n* = 15 for saline and *n* = 14 for both 0.5 mg/kg and 1.0 mg/kg fingolimod as three mice were euthanised during surgery, two mice with no lesion were excluded, and one mouse was excluded due to a damaged histological sample (details on excluded mice are shown in Supplementary Figure S3). All treatment groups had a significant weight loss after stroke that did not significantly improve until day 7 when compared to day 1 after stroke (saline *p* = 0.018, 0.5 mg/kg *p* = 0.003, 1.0 mg/kg *p* = 0.033) (Supplementary Figure S4A).

**FIGURE 2:**
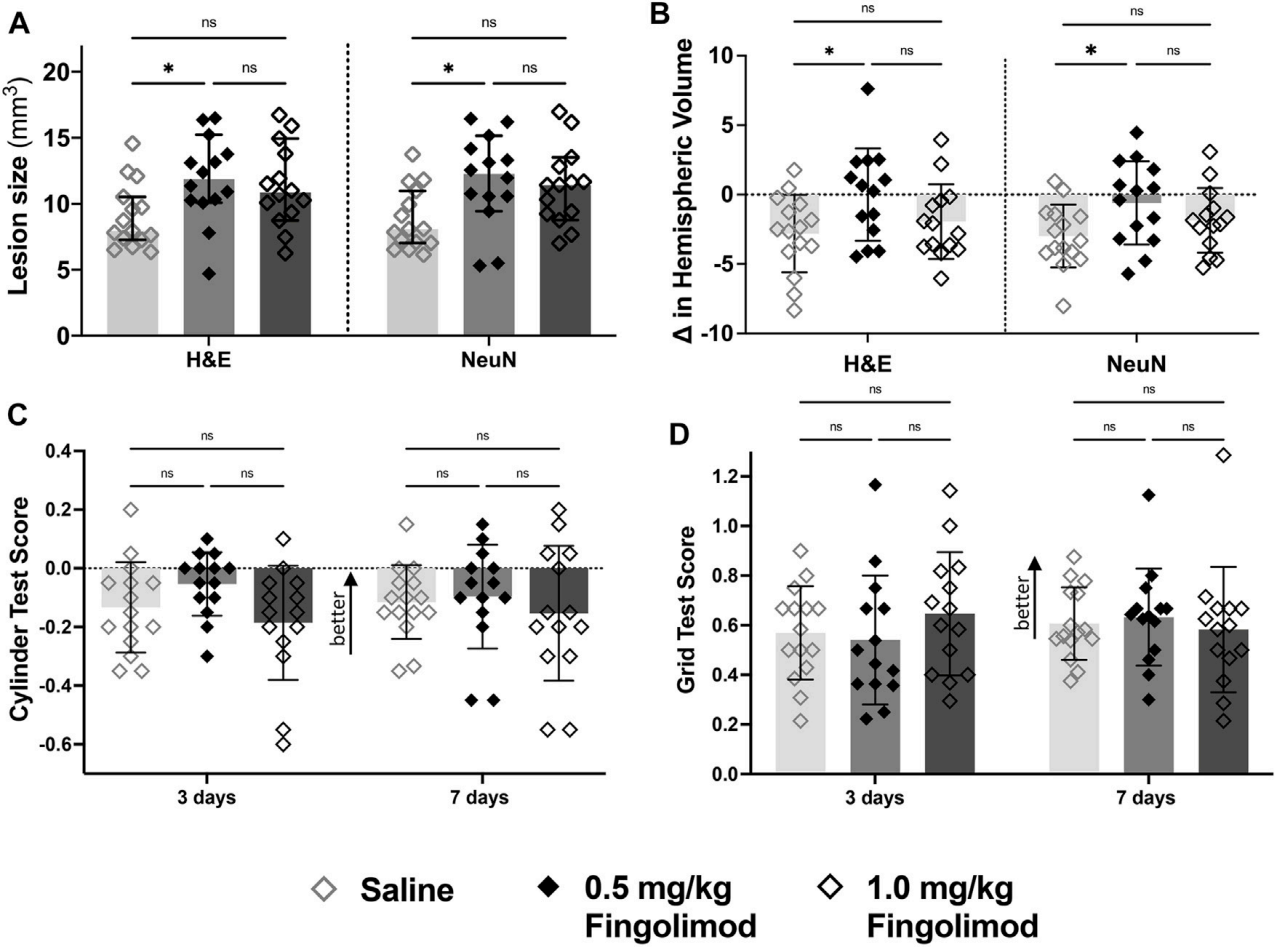
Dose response study—**(A)** Lesion size measurements, by staining method; saline-treated mice had a smaller lesion size 7 days after stroke compared to the 0.5 mg/kg dose for both staining methods. **(B)** Difference in volume between brain hemispheres; saline-treated mice had a significant reduction in the hemispheric volume (L-R/SUM) compared to the 0.5 mg/kg group. **(C)** Cylinder test results at 3- and 7-days post-stroke (negative scores represent preference for the left paw usage (ipsilateral)); there was no difference in scores over time. **(D)** Grid walking test scores (scores closer to 1 represent a more comparable number of missed steps on each side); there was no difference over time or between treatment groups ◇ Saline (*n* = 15) ◆ 0.5 mg/kg fingolimod (*n* = 14) ◇ 1.0 mg/kg fingolimod (*n* = 14). Mean ± SD; ns - not significant; *p < 0.05.

Lesion size comparison in both H&E- and NeuN-stained sections revealed a significant increase between saline and the lower dose of fingolimod (H&E *p* = 0.033; NeuN *p* = 0.022) (Figure 2; representative sections illustrating lesion size and location are shown in Supplementary Figure S5). There was no difference between the 1 mg/kg fingolimod group and both saline and the lower dose of fingolimod (H&E: saline vs. 1.0 mg/kg *p* = 0.11, 0.5 mg/kg vs. 1.0 mg/kg *p* > 0.999; NeuN: saline vs. 1.0 mg/kg *p* = 0.073, 0.5 mg/kg vs. 1.0 mg/kg *p* > 0.999). At variance with the increased infarct size, 0.5 mg/kg fingolimod (H&E *p* = 0.042; NeuN *p* = 0.040), but not the higher dose (H&E *p* > 0.999; NeuN *p* = 0.46), attenuated the ipsilateral deficit in hemisphere volume.

The cylinder test revealed no interaction between time and treatment and no difference between treatment groups 3 or 7 days after stroke (interaction *p* = 0.24, time *p* = 0.32, treatment *p* = 0.92). The foot fault test revealed an effect of time (*p* < 0.001) but not treatment or the interaction of the variables (*p* = 0.708, *p* = 0.777), suggesting that neither dose of fingolimod had an impact on the functional recovery following stroke.

### 3.2 Aged Mice

Two mice were euthanised during surgery, and three mice, all in the saline group, had to be euthanised after reaching the threshold score for humane endpoint at days 2, 4, and 5 post-stroke (Supplementary Figure S6). A Log-rank test of the survival curve revealed that there was only a trend for improved survival in fingolimod-treated mice (*p* = 0.073). Two mice were excluded from the analysis, one in each treatment group, because the brain sections showed bleeding. The final number of mice in the saline and fingolimod groups were *n* = 11 and *n* = 14, respectively.

Mice from both treatment groups had significantly lower weight post-surgery compared to the start of the study. Weight loss was more protracted in saline-treated mice, who showed continued weight loss beyond day 4, whereas the weight of fingolimod-treated mice trended back up (Supplementary Figure S4B).

**FIGURE 3:**
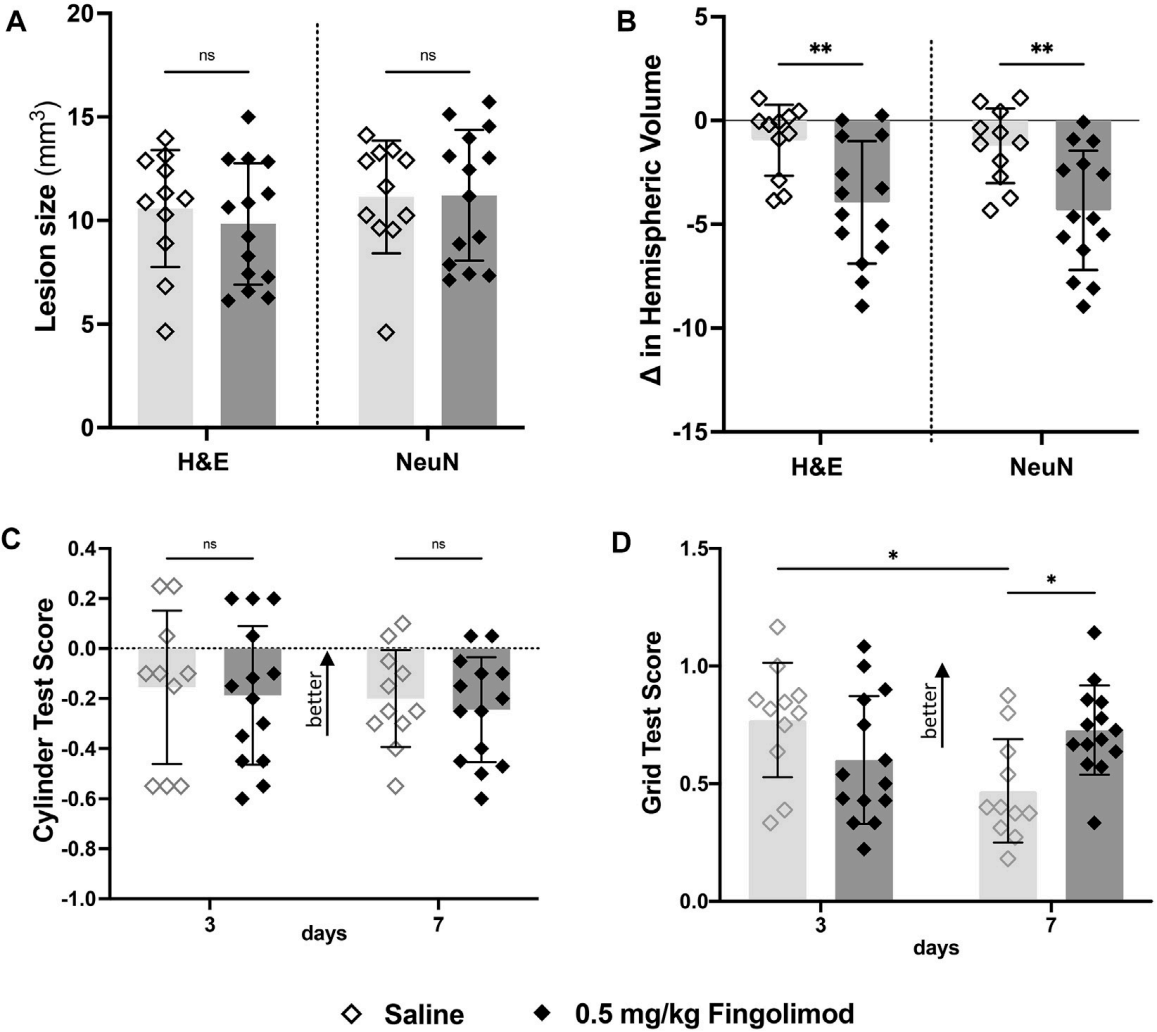
Aged mouse study—**(A)** Lesion size measurements, by staining method; there was no difference in lesion size 7 days after stroke between treatments for both staining methods. **(B)** Difference in volume between brain hemispheres; fingolimod (0.5 mg/kg) treated mice had a significantly reduced ipsilateral hemispheric volume compared to saline. **(C)** Cylinder test results at 3- and 7-days post stroke by treatment group (negative scores represent preference for the left paw usage (ipsilateral)); there was no difference in scores over time. **(D)** Grid walking test scores (scores closer to 1 represent a more comparable number of missed steps on each side); saline-treated mice scored significantly worse than their day 3 scores, mice treated with fingolimod did not differ in their scores. ◇Saline (*n* = 11) ◆ 0.5 mg/kg fingolimod (*n* = 14). Mean ± SD; ns - not significant; *p < 0.05; **p < 0.005; ***p < 0.0005.

Lesion sizes did not significantly differ between saline- and fingolimod-treated mice (Figure 3A). However, there was a significant treatment-related difference between the hemispheric volume deficits, with the lower dose of fingolimod showing a larger deficit compared to the saline-treated mice when measured in both H&E- (*p* = 0.004) and NeuN- (*p* = 0.003) stained samples (Figure 3B).

The cylinder test did not show any treatment-related differences (time *p* = 0.083; treatment *p* = 0.65; interaction *p* = 0.99) (Figure 3C). In contrast, foot fault scores (Figure 3D) revealed a significant interaction between time and treatment variables (*p* = 0.005), but not time (*p* = 0.23) or treatment independently (*p* = 0.49). Bonferroni post-hoc multiple comparisons showed that the performance of saline-treated mice deteriorated between 3 and 7 days (*p* = 0.017), while fingolimod-treated mice performed better than saline at day 7 (*p* = 0.017).

### 3.3 Atherosclerotic Mice

Seven mice were euthanised during the surgical procedure. The remaining mice were allocated randomly to the saline (*n* = 16) or 0.5 mg/kg fingolimod groups (*n* = 15). Following stroke, mice in both treatment groups lost weight, but there was no difference between the groups (Supplementary Figure S4C).

**FIGURE 4:**
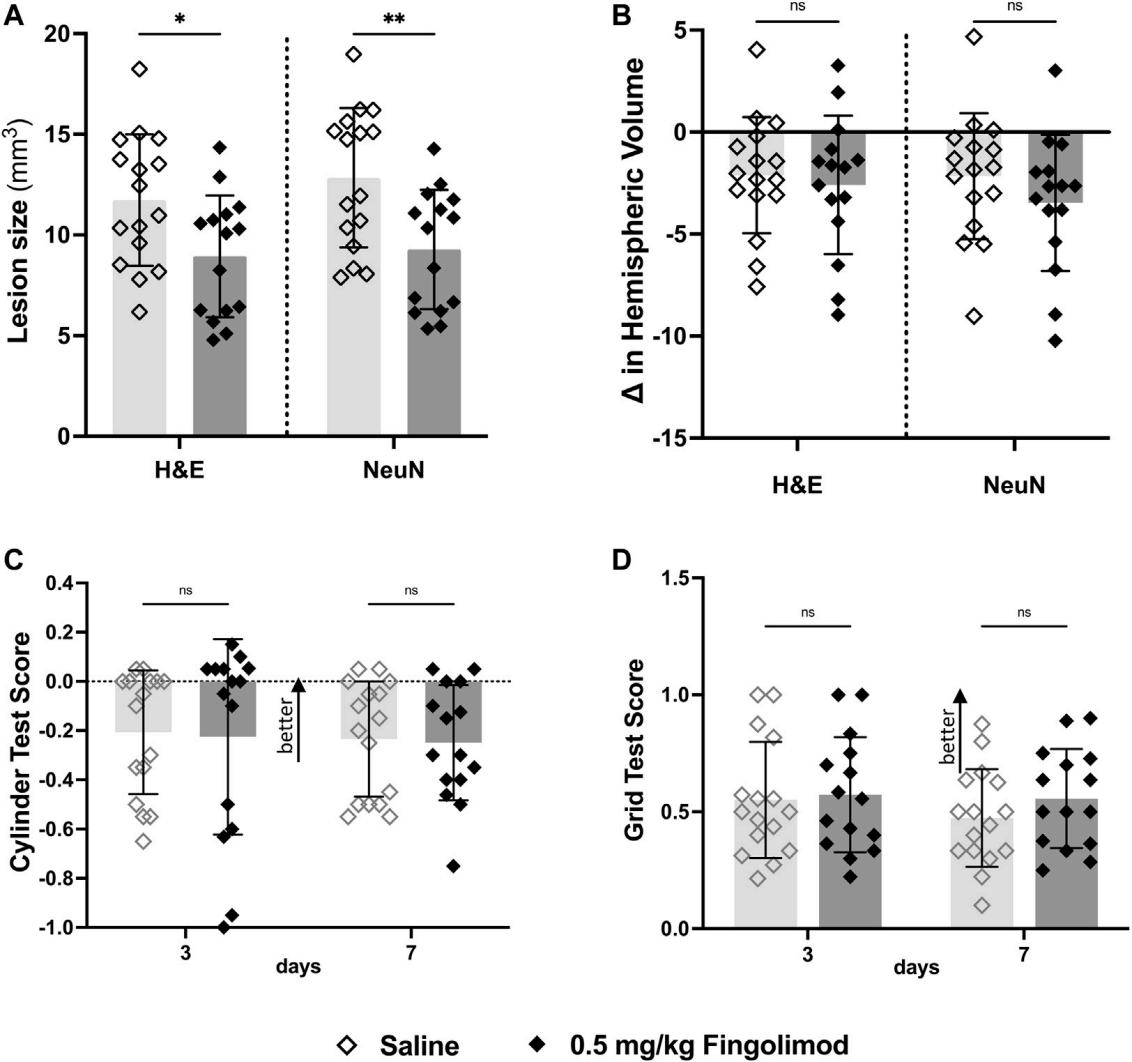
Atherosclerotic mouse study**—(A)** Lesion size measurements by staining method; fingolimod (0.5 mg/kg) treated mice had a smaller lesion size 7 days after stroke compared to saline for both staining methods. **(B)** Difference in volume between brain hemispheres; there was no difference in the hemispheric volumes between treatment groups **(C)** Cylinder test results at 3- and 7-days post-stroke arranged by treatment group (negative scores represent preference for the left paw usage (ipsi)); there was no difference in scores over time. **(D)** Grid walking test scores (scores closer to 1 represent a more comparable number of missed steps on each side) to there was no difference over time or between treatment groups ◇Saline (*n* = 16) ◆ 0.5 mg/kg fingolimod (*n* = 15). Mean ± SD; ns - not significant; *p < 0.05; **p < 0.005; ***p < 0.0005.

Fingolimod-treated mice had a smaller lesion size than saline-treated mice (H&E: *p* = 0.02; NeuN: *p* = 0.005) (Figure 4A). Ipsilateral hemispheres showed small atrophy in all groups, with no effect of fingolimod (H&E: *p* = 0.673; NeuN: *p* = 0.266) (Figure 4B).

The cylinder test (Figure 4C) revealed no interaction between time and treatment (*p* = 0.970), and no independent effect of time or treatment (*p* = 0.6678, *p* = 0.841). The foot fault test (Figure 4D) did not show interaction between time and treatment (*p* = 0.77), and no effect of time (*p* = 0.76), or treatment (*p* = 0.25).

### 3.4 Treatment Duration

A total of 51 mice were used for this study, three of which were euthanised during surgery. The remaining 48 mice were randomly allocated to the treatment groups (*n* = 16 per group). All groups lost weight after stroke, with no effect of fingolimod (Supplementary Figure S4D).

**FIGURE 5:**
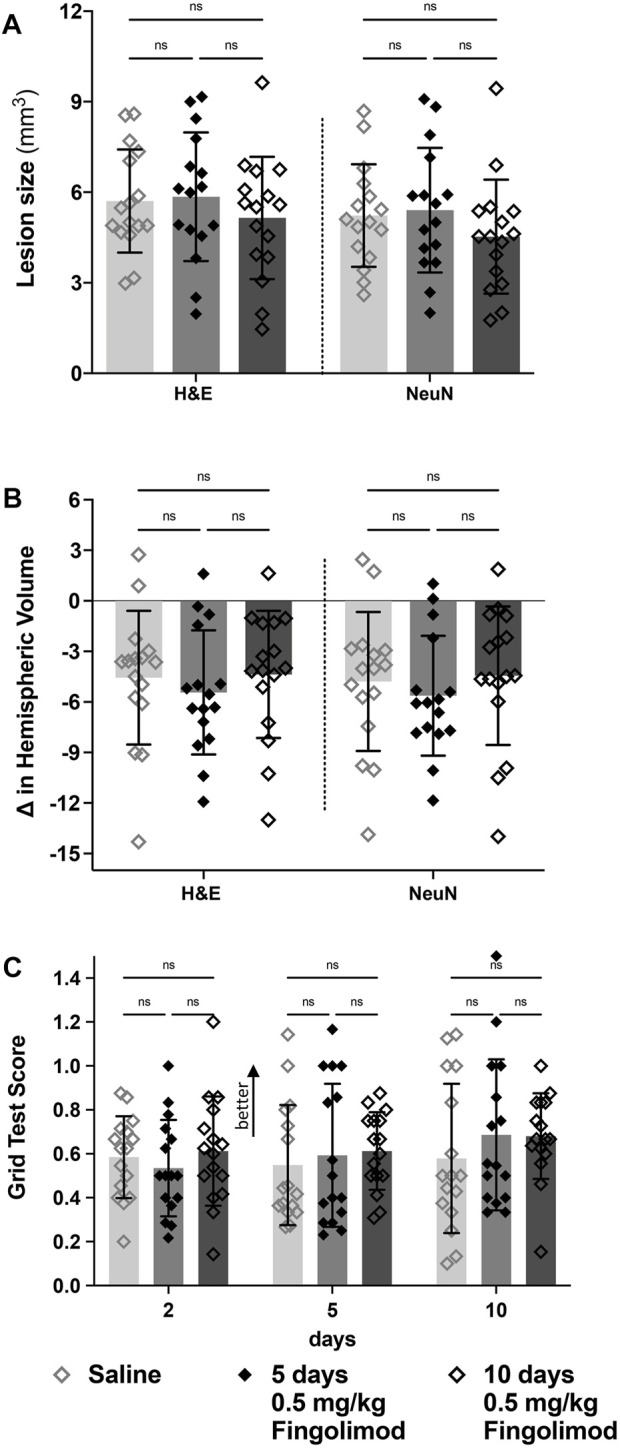
Treatment duration study—**(A)** Lesion size measurements, by staining method; there was no difference in the lesion size between saline, 5- and 10-days of fingolimod treatment. **(B)** Difference in volume between brain hemispheres; there was no difference in the hemispheric volumes between treatment groups. **(C)** Grid walking test scores (scores closer to 1 represent a more comparable number of missed steps on each side); there was no difference over time or between treatment groups ◇Saline (*n* = 16) ◆ 0.5 mg/kg fingolimod for 5 days (*n* = 16) ◇ 0.5 mg/kg fingolimod for 10 days (*n* = 16). Mean ± SD; ns - not significant; *p < 0.05; **p < 0.005; ***p < 0.0005.

Five or 10 days of fingolimod did not reduce lesion sizes compared to saline-treated mice (Figure 5A). No differences were observed when evaluating the change in ipsilateral hemispheric volume (NeuN: saline vs. 5 days *p* = 0.89, saline vs. 10 days *p* = 0.99, 5 days vs. 10 days *p* = 0.78) (Figure 5B).

In the foot fault test (Figure 5C), there was no significant effect of time (*p* = 0.291), treatment (*p* = 0.585) or interaction between these variables (*p* = 0.79).

### 3.5 Effect of Fingolimod on T Cell Distribution

**FIGURE 6:**
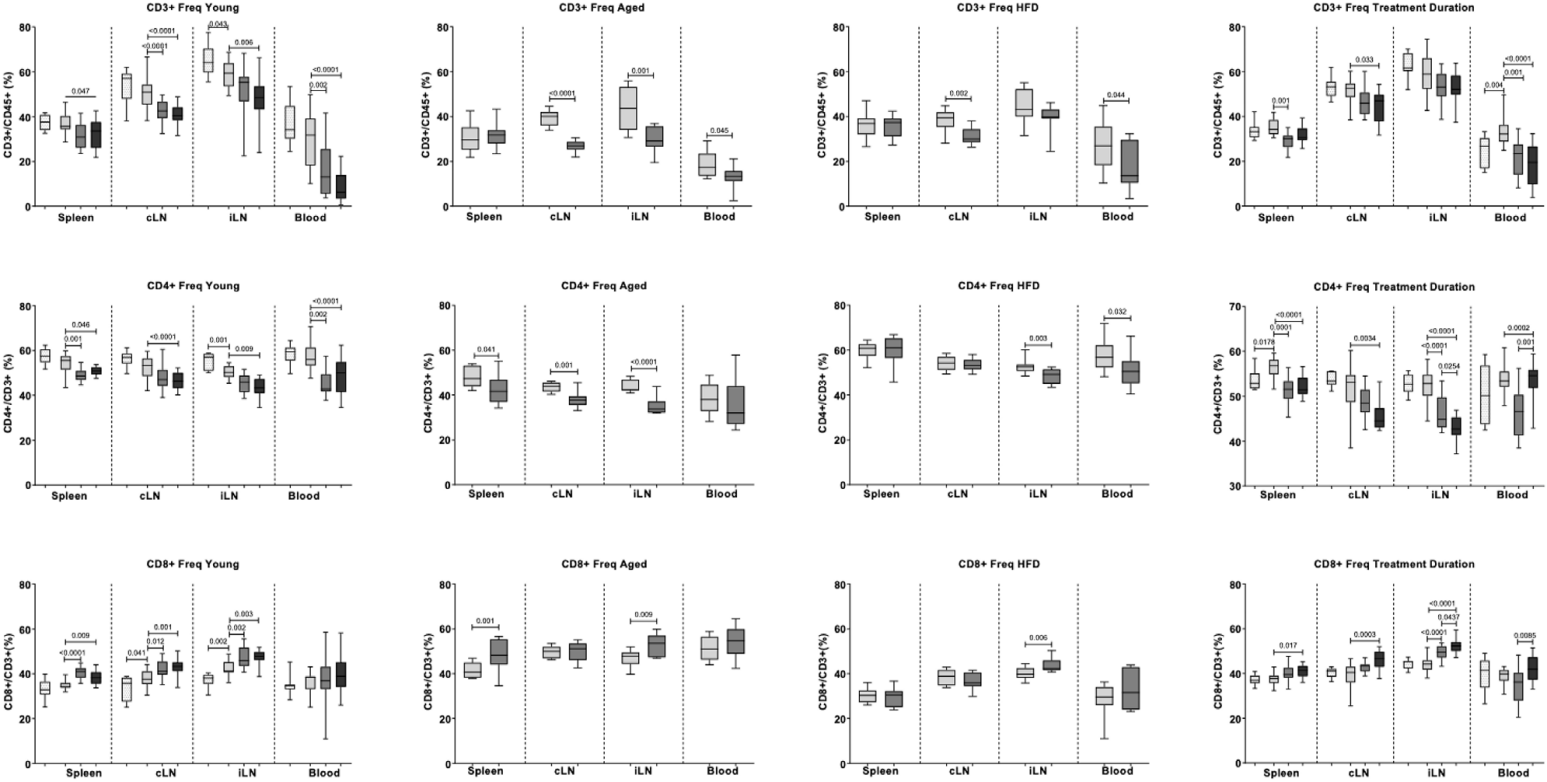
Effects of fingolimod on the frequencies of CD3^+^, CD4^+^, and CD8^+^ cells in blood, cervical lymph nodes (cLN), inguinal lymph nodes (iLN) and spleen. Values obtained in the Dose Response, Aged, Atherosclerosis (High fat diet, or HFD) and Treatment Duration studies are shown as medians and confidence intervals (empty bars: naïve mice; light grey bars: saline-treated mice; middle and dark grey bars: 0.5 mg/kg and 1 mg/kg fingolimod-treated mice). Two-sided, independent-samples t tests assessed differences between two groups. One-way analysis of variance (ANOVA) tests with post hoc Tukey’s multiple comparisons were used to investigate differences between three or more groups. *p* values are shown above the relevant comparison.

In young mice (dose-response study), stroke reduced T cell frequency (CD3^+^) in inguinal lymph nodes (Figure 6). Both fingolimod doses reduced T cell frequency in blood (*p* < 0.0001) and cervical lymph nodes (*p* < 0.0001). In inguinal lymph nodes, only 1 mg/kg fingolimod reduced T cell frequency (*p* = 0.005).

In aged and ApoE−/− cohorts, fingolimod (0.5 mg/kg) did not affect CD3^+^ cell frequency in spleen (Figure 6). Fingolimod decreased T cell frequency in blood (Aged: *p* = 0.045, ApoE−/−: *p* = 0.043), draining lymph nodes (Aged: *p* < 0.0001, ApoE−/−: *p* = 0.001) and non-draining lymph node, but only in aged mice (*p* = 0.001).

In young mice, stroke decreased CD4^+^ cell frequency in non-draining lymph nodes (*p* = 0.001) (Figure 6). No statistically significant differences were observed in other tissues. Low dose fingolimod decreased CD4^+^ cell frequency in spleen (*p* = 0.001), blood (*p* = 0.002), and non-draining lymph nodes (*p* = 0.001). Similar effects were observed with high dose fingolimod, with reduced CD4^+^ cell frequency in spleen (*p* = 0.046), blood (*p* < 0.0001), draining lymph nodes (*p* = 0.009), and non-draining lymph nodes (*p* < 0.0001).

In aged mice, low-dose fingolimod reduced CD4^+^ cell frequency in spleen (*p* = 0.041), draining lymph nodes (*p* = 0.001), and non-draining lymph nodes (*p* < 0.0001). In ApoE−/− mice, a decreased CD4^+^ cell frequency was only recorded in blood (*p* = 0.032) and non-draining lymph nodes (*p* = 0.003).

In young mice, stroke increased CD8^+^ cell frequency in draining (*p* = 0.041) and non-draining (*p* = 0.002) lymph nodes. Low-dose fingolimod increased CD8^+^ cell frequency in draining lymph nodes (*p* = 0.012), non-draining lymph nodes (*p* = 0.002), as well as spleen (*p* < 0.0001). A similar increase in CD8^+^ cell frequency was observed in the draining lymph nodes (*p* = 0.001), non-draining lymph nodes (*p* = 0.003), and spleens (*p* = 0.009) of mice that received the higher dose of the drug.

In aged mice, fingolimod increased CD8^+^ cell frequency in spleen (*p* = 0.001) and non-draining lymph nodes (*p* = 0.009). In ApoE−/− mice, an increased CD8^+^ cell frequency was only recorded in non-draining lymph nodes (*p* = 0.006).

Previously published immunohistochemical analysis performed in the brains of mice used in the current study showed that both doses of fingolimod decreased the number of CD3^+^ cells in the ischemic core, while only 0.5 mg/kg showed this effect in the peri-infarct area (Malone et al., 2021). This dose of fingolimod also decreased the number of infiltrating CD3^+^ cells in the peri-infact area of aged and ApoE−/− mice, with a trend to a decrease, but no statistical significance, in the infarct core.

### 3.6 Correlation Between T Cell Frequencies and Post-stroke Outcome Measures

As three studies (Dose Response, Aged, Atherosclerotic mice) employed the same treatment protocol, combined stroke outcome and flow cytometry data were examined for any correlation between lymphocytes and experimental outcomes in a large heterogeneous population of saline and fingolimod-treated mice and of mice in both treatment groups.

**FIGURE 7:**
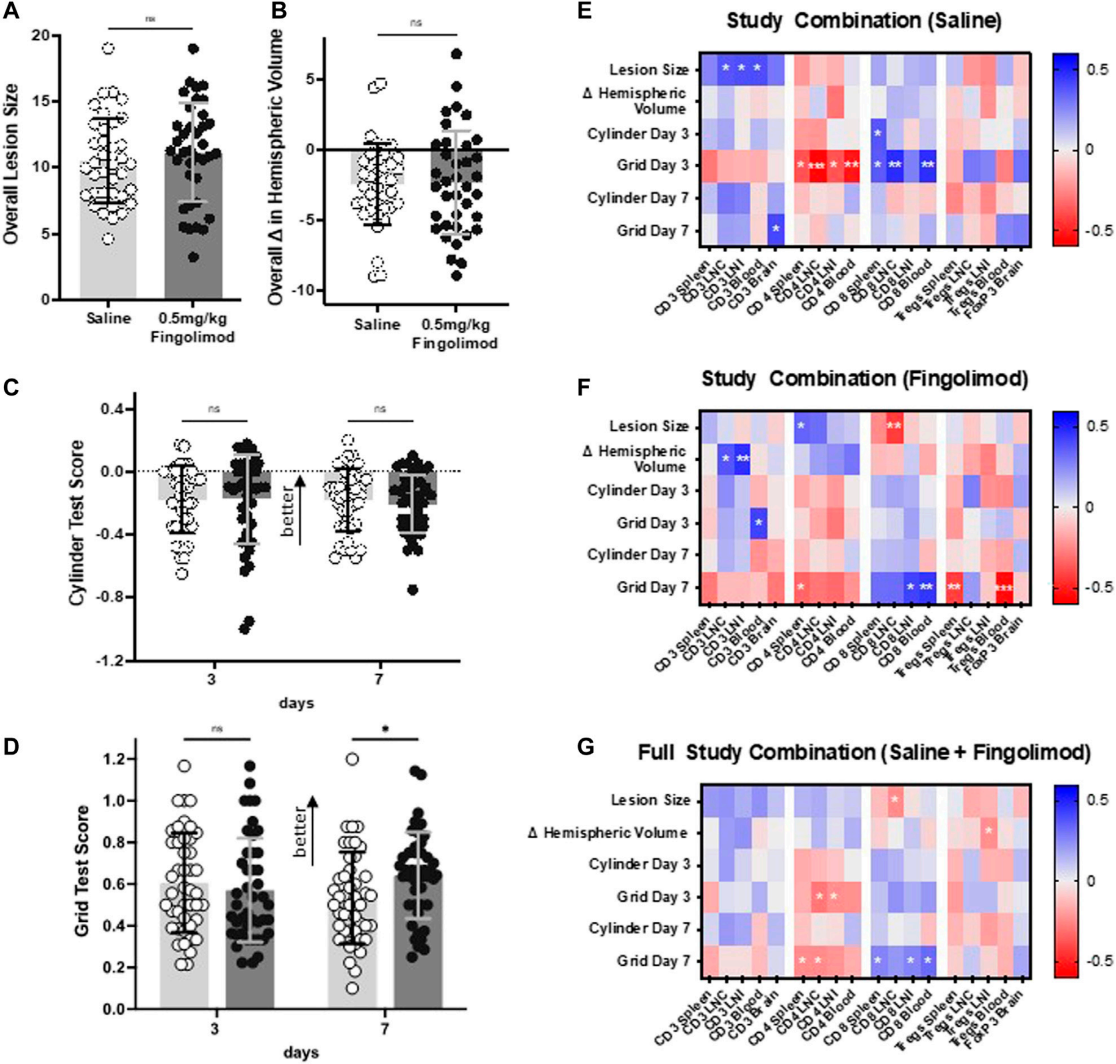
Results from combined studies (dose response, aged, atherosclerotic mice) showing effect of daily saline or 0.5 mg/kg fingolimod on **(A)** lesion size, **(B)** difference in hemispheric volumes, **(C)** cylinder test results, **(D)** grid walking test score (*n* = 37 per drug treatment group). **E–G**: Correlation matrix between the proportion of T lymphocytes and experimental outcomes. Heat map depicting correlations between lymphocyte proportions and experimental outcomes (infarct volume, hemispheric volume, and cylinder/foot fault scores at days 3 and 7) for **(A)** all young, aged, and ApoE^−/−^ mice, **(B)** saline-treated mice, and **(C)** fingolimod-treated mice. Colours represent Pearson r value on a scale from −0.6 to +0.6, as indicated on the lookup tables on the rights. For statistical significance, *p < 0.05, **p < 0.01, ***p < 0.001.

Pooled outcome measures (*n* = 37 per group) showed that 0.5 mg/kg fingolimod treatment decreased the number of foot fault in the grid test at 7 days (*p* = 0.029) (Figure 7D). This treatment had no effect on lesion size (Figure 7A), hemispheric deficit (Figure 7B), or cylinder test scores (Figure 7C).

The correlation analysis between outcome measures and flow cytometry data is shown as heat maps in Figures 7E–G. Examples of correlations are illustrated in Supplementary Figure S7. In saline-treated mice, CD4^+^ cells in spleen (*p* = 0.024), draining lymph nodes (LNC) (*p* = 0.0004), non-draining lymph nodes (LNI) (*p* = 0.024), and blood (*p* = 0.001) showed a significant negative correlation with behavioural scores as assessed by grid-walking test at day 3 post-ischaemia. Conversely, CD8^+^ cells in spleen (*p* = 0.033), draining lymph nodes (*p* = 0.004), and blood (*p* = 0.006) displayed a positive correlation (Figure 7E). In fingolimod-treated mice these correlations were mostly apparent 7 days post-ischaemia, with CD4^+^ cells in spleen (*p* = 0.046) showing significant negative correlations, while CD8^+^ cells in non-draining lymph nodes (*p* = 0.011) and blood (*p* = 0.006) displayed positive correlations (Figure 7F). In saline-treated mice, regulatory T cells (Tregs) showed a weak positive correlation with 7 days recovery in the grid-walking test (e.g., blood: *p* = 0.144). Post-fingolimod treatment however, Tregs in spleen (*p* = 0.009) and blood (*p* = 0.0005) displayed negative correlations with 7 days grid-walking test scores.

An analysis of the combined data from saline-treated and fingolimod-treated mice (Figure 7G) shows that this treatment effect was still evident at 7 days, with CD4^+^ cells in spleen (*p* = 0.0430) and draining lymph nodes (0.037) negatively correlated with recovery, whereas CD8^+^ cells in spleen (*p* = 0.028), non-draining lymph nodes (*p* = 0.027), and blood (*p* = 0.006) showed significant positive correlations.

## 4 Discussion

The objectives of these studies were to test the hypothesis that fingolimod is effective in a large, statistically well-powered experimental ischemic stroke study, in animal models relevant to the clinical features of stroke patients, with clinically relevant experimental outcome measures. The primary positive findings of this report are that: i) treatment with 0.5 mg/kg fingolimod, led to histological improvement in mice with hyperlipidaemia, but showed inconsistent effects on histological parameters (infarct size and tissue atrophy) in young mice ii) behavioural improvements were seen only in aged mice, and when mice from all studies were pooled. Trends of a beneficial effect of fingolimod were also observed in the survival rate of aged mice. Although weight change was not a pre-planned outcome measure, and studies were not specifically powered to detect a treatment effect on this variable, weights were recorded in all mice. Except for a trend to a faster weight recovery in fingolimod-treated aged mice, fingolimod generally had no effect on this outcome measure, further indicating that the drug was not effective under the conditions of our studies. In contrast with these few positive effects on post-stroke histological and behavioural improvement, fingolimod robustly reduced the frequencies of lymphocyte populations (CD3^+^, CD4^+^) in blood and various lymphoid tissues.

This preponderance of neutral effects on stroke outcome measures contrasts with the generally positive effects of fingolimod in published studies (Liu et al., 2012; Dang et al., 2020). A meta-analysis concluded that fingolimod reduced infarct volume by 30.4% and improved behavioural outcome by 34.2% (Dang et al., 2020). Another study documenting an effect of fingolimod was published afterwards (Wang et al., 2020). Of 18 published studies, only three demonstrated no significant effect of fingolimod. One involved a filament-induced 3-h MCA occlusion in normal young mice (Cai et al., 2013), another involved 1-h filament MCA occlusion on diabetic mice (Li et al., 2020), and the third used distal MCA electrocoagulation (Liesz et al., 2011). The fact that the latter study and our study used the same permanent distal model, and both yielded non-significant results suggests that reperfusion and/or subcortical involvement is required for fingolimod to show benefits. However, fewer infiltrating leukocytes are found 24 h after transient MCA occlusion compared with permanent MCA occlusion (Chu et al., 2013). Furthermore, previous studies documented a beneficial effect of fingolimod in the absence of reperfusion (Wei et al., 2010; Campos et al., 2013). The fact that one of these studies also used a permanent photothrombotic stroke model with no subcortical involvement argues against the possibility that the effectiveness of fingolimod requires both reperfusion and large infarcts (Brunkhorst et al., 2013).

Fingolimod reduces hepatic ischemia/reperfusion injury by reducing T cell infiltration (Martin et al., 2009). The immunosuppressive effects of fingolimod in multiple sclerosis are also well documented (Brinkmann, 2009; Kappos et al., 2010). In experimental stroke, the effects of fingolimod on circulating lymphocytes were assessed in six studies, including two that failed to show a treatment effect on stroke outcome (Liesz et al., 2011; Cai et al., 2013). The protective effect of fingolimod is not observed in lymphocyte-deficient Rag1^−/−^ mice. These studies suggest that fingolimod-induced lymphocytopenia is necessary, but may not be sufficient, for its stroke-protective action (Kraft et al., 2013). This is confirmed by the observation that 0.5 mg/kg fingolimod decreased the number of infiltrating lymphocytes (CD3^+^ cells) in the peri-infarct area of the young, aged and hyperlipidaemic mice used in the current study (Malone et al., 2021).

To increase the external validity of our studies (Voelkl et al., 2021) and mimic the heterogeneous population that would be enrolled in a clinical trial, we analysed the pooled data obtained with 0.5 mg/kg fingolimod from the dose-response study together with data from the aged and atherosclerotic mice. Only the improvement in the grid walking test at 7 days showed a significant treatment effect, suggesting that this test may be a more robust predictor of functional improvement when stroke only affects the sensorimotor cortex. Based on this observation and to further test the hypothesis that immunosuppression plays a role in recovery, we examined the correlation between the profiles of various T cell populations in the periphery and our outcome measures. In the absence of fingolimod, there were strong correlations between the frequencies of T cells in the periphery (negative correlation for the frequency of CD4^+^ cells and positive for CD8^+^ cells) and improved performance in the grid walking test at 3 days. Surprisingly, fingolimod treatment weakened these correlations at 3 days, but markedly strengthened the correlation between lymphocytes frequencies and performance in the grid walking test at 7 days. While other correlations were weaker, heat map analysis in fingolimod-treated mice showed a generally negative correlation between peripheral CD4^+^ cells and improved behavioural outcome at both 3 and 7 days, whereas the correlation with CD8^+^ cells was generally positive. Relationships were inverted when considering the associations between histological improvement and peripheral CD4^+^ and CD8^+^ frequencies.

While this does not establish causality or mechanisms, these observations suggest that CD4^+^ and CD8^+^ cells might play opposing roles in histological and behavioural recovery, possibly because infarct size responds to mechanisms protecting the penumbra early after stroke (Liu et al., 2010). In contrast, improved behavioural outcomes may be mediated by effects on inflammation-driven secondary injury (Iadecola and Anrather, 2011) or neuronal plasticity prevailing later in the pathophysiological cascade (Regenhardt et al., 2020). Based on this hypothesis and because fingolimod improved behavioural recovery in pooled data, we examined whether extending fingolimod treatment to 10 days resulted in more clear-cut improvement. These studies showed no effect, indicating that the lack of improvement following fingolimod treatment is related to the mouse model and/or study conditions, rather than an inappropriate treatment regimen. Our results therefore contrast with data showing beneficial effects of fingolimod in a well-powered experimental stroke study likely to emphasize repair mechanisms, in which it was administered for 5 days starting on post-stroke day 3 (Brunkhorst et al., 2013).

Following the discovery that CD4^+^ or CD8^+^-deficient mice show improved outcome as early as 24 h after reperfusion (Yilmaz et al., 2006), numerous studies have shown the deleterious role of T-lymphocytes in experimental stroke (Malone et al., 2018). Pharmacological interventions targeting these cells are effective in experimental models (Malone et al., 2019). Among these, the multiple sclerosis agent natalizumab progressed to stroke clinical studies after showing efficacy in four preclinical studies (discussed in Llovera et al., 2015) and despite an experimental stroke study with neutral results (Langhauser et al., 2014). The phase II ACTION trial showed that natalizumab did not affect infarct volume but improved functional outcomes at 30 days, but not at 90 days (Elkins et al., 2017). The follow-up ACTION-II trial, however, did not meet its primary or secondary endpoints (Elkind et al., 2020). A multicenter preclinical randomized controlled trial showed that anti-CD49d treatment showed modest neuroprotection after permanent distal MCA occlusion (the model used in the present study), but no effect in a 1 h MCA occlusion, which causes more widespread damage (Llovera et al., 2015). This difference was tentatively attributed to the higher number of brain leukocytes, microgliosis, and proinflammatory cytokine release observed in the distal model. It also further argues against the possibility that the lack of effect of fingolimod in the present and a previous study is accounted for by their common use of distal MCA electrocoagulation (Liesz et al., 2011).

Aging and atherosclerosis are associated with a pro-inflammatory state that may be detrimental to stroke outcome (Franceschi et al., 2018; Bäck et al., 2019). The total number of CD4^+^ and CD8^+^ T cell populations are lower in older individuals (Ahnstedt and McCullough, 2019). Although atherosclerotic patients are usually older, they show relatively higher proportions of both CD4^+^ and CD8^+^ T cells compared with younger individuals (Neupane et al., 2019). We therefore tested fingolimod in aged and in atherosclerotic mice. Both mouse cohorts showed a larger infarct size than young mice. Aged mice showed functional but not histological fingolimod-induced improvement, while atherosclerotic mice showed the opposite treatment effect. These results suggest that, in aged mice, fingolimod may be more effective on processes that mediate neuronal plasticity, likely to occur later in the pathophysiological cascade, while early cytoprotective mechanisms may predominate in atherosclerotic mice.

In a parallel study performed on the same cohort of mice, we observed that fingolimod increased to a larger extent the numbers of CD4^+^CD25^+^Foxp3^+^ Treg lymphocytes in the brain of aged mice compared to the brains of young or atherosclerotic mice (Malone et al., 2021). This suggests that, following stroke, Tregs may play a prominent protective role in aged mice, in agreement with the observation that mouse and human Tregs increase in number and function with aging (Sharma et al., 2006; Rosenkranz et al., 2007).

Pro-inflammatory effects of aging and atherosclerosis contribute to the poorer functional recovery in animal models of these disorders (Hermann et al., 2019), and a systemic immune-inflammation index is associated with stroke severity and recovery in stroke patients (Weng et al., 2021). It is possible that more subtle differences in inflammatory and/or immune status may explain why fingolimod is effective in most but not all experimental stroke studies. Housing conditions have been shown to affect the progression of obesity-related insulin resistance and adaptive immunity, with ‘‘antigen exposed’’ mice showing more effector memory T cells and lower percentages of naïve T cells compared to mice housed under specific pathogen-free (SPF) conditions (Sbierski-Kind et al., 2018). Differences in intestinal microbiome composition regulate the Th17:Treg balance in the small intestine (Ivanov et al., 2008) and affect short- and long-term outcomes after ischemic stroke (Benakis et al., 2020). Based on these observations, it is tempting to speculate that different housing conditions or microbiota may account for the lack of effects of fingolimod in this and previous studies, including studies of haemorrhagic stroke (Diaz Diaz et al., 2020).

This study examined the effects of fingolimod on T cell frequency, based on the hypothesis that the effects of this agent on lymphocytes mediate its beneficial effects in stroke (Kraft et al., 2013). It should however be noted that fingolimod acts on 4 of the 5 S1P receptor subtypes (Brinkmann et al., 2010), and direct effects on other cell types may also contribute. These pleiotropic effects were recently reviewed in the context of multiple sclerosis and may also be relevant in stroke (Colombo and Farina, 2022). Effects on the vasculature are particularly important for stroke outcome. We have previously shown that 0.5 mg/kg or 1 mg/kg fingolimod does not acutely affect cerebral blood flow when administering the drug after MCAO (Wei et al., 2010). More recently, fingolimod (2 × 5 mg/kg, orally) was shown not to activate endothelial S1P_1_ receptors, possibly due to the abluminal localization of these receptors (Nitzsche et al., 2021). In apparent contrast with this lack of effect on brain endothelium, fingolimod reduces hemorrhagic transformation associated with delayed tissue plasminogen activator treatment in a mouse thromboembolic model (Campos et al., 2013). While fingolimod was only administered for 2 days in the latter study, 35 days fingolimod administration decreases endothelial S1P_1_ receptors, increases systemic blood pressure and induces vascular dysfunction in control mice, suggesting that long-term treatment might be detrimental (Cantalupo et al., 2017). Our attempt to extend the treatment duration did not show any benefit or detrimental effect, but 10 days may have been too short to observe negative effects. The clinical significance of these effects of fingolimod on vascular function is unclear, as long-term fingolimod treatment rarely affects blood pressure in patients (Paolicelli et al., 2015).

Discrepancies in the literature relating to fingolimod in experimental stroke studies may also be related to differences in their quality. Liu et al. (2012) and Dang et al. (2020) reveal large differences in the rigour with which preclinical studies were conducted. Only 6 of 17 publications reported the use of blinded allocation, and four publications reported using *a priori* power calculations. As only a quarter of the studies used group sizes larger than 10, it is likely that most studies were underpowered, possibly leading to false positive and/or overestimated treatment effect sizes (Button et al., 2013). Of note, while our study was based on power calculation using the standard deviation of infarct sizes in preliminary study, the observed variability of our behavioural data was larger than anticipated, and our conclusion may also rely on studies with a relatively low power. Another limitation of our study is that the doses of 0.5 and 1 mg/kg were selected from studies performed on young animals. The dose of fingolimod for the aged and hyperlipidaemic mice might not be optimal due to potential differences in drug pharmacokinetics in aged animals with comorbidities.

The current studies were based on *a priori* group size calculations, with no correction for multiple comparisons. Therefore, it is possible that the few positive effects we found resulted from type I errors. In addition to this limitation, we chose not to include female mice in the study. Studies of fingolimod in experimental ischemic stroke were all done in males, and we failed to replicate effects of fingolimod in experimental haemorrhagic stroke using both male and female mice (Diaz Diaz et al., 2020). Local factors such as breeder, substrain and microbiota, rather than sex differences may therefore explain the lack of treatment effect. Finally, we only monitored the body temperature, not blood pressure or blood gases. Accurately measuring these physiological parameters can only be done invasively and would have interfered with functional outcome. However, previous studies found no effect of fingolimod on post-MCA occlusion cerebral blood flow, heart rate, blood pressure, blood gases, and temperature (Wei et al., 2010).

In conclusion, fingolimod may only be effective in animals with specific inflammatory and immune profiles. Should future studies show that fingolimod is truly effective, but only in animals with a defined microbiome, the challenge will be to determine how to translate these findings clinically. Importantly, this issue may not be unique to fingolimod, but may affect other drugs acting as immunomodulators. Natalizumab showed similarly discrepant results across preclinical studies and has failed to show clinical benefits. Only small stroke trials have been conducted with fingolimod. While three open-label studies in patients with ischemic stroke (Fu et al., 2014; Zhu et al., 2015; Tian et al., 2018) showed encouraging effects, these results await confirmation in larger trials. Despite the number of studies of fingolimod in experimental stroke and the few clinical studies, the efficacy of this agent in stroke is not settled. While there is a preponderance of studies showing a significant effect, the fact that our study only shows positive effects on some outcome measures in specific mouse populations suggests that additional variables may act as a confounding factors, at least in rodents. Understanding the nature and the role of these variables may inform the design of future clinical trials and may be the key to the discovery of elusive neuroprotective agents.

## Data Availability

The datasets presented in this study can be found in online repositories. The names of the repository/repositories and accession number(s) can be found below: https://data.mendeley.com/datasets/ctyx59p5rh/1 (Mendeley Data).
