# Supplementary material for: Preclinical Evaluation of Fingolimod in Rodent Models of Stroke With Age or Atherosclerosis as Comorbidities
Source: Front Pharmacol. 2022 Jul 13;13:920449. doi: 10.3389/fphar.2022.920449 (PMC9326401; doi:10.3389/fphar.2022.920449)
Supplement: Supplementary file 1 [file DataSheet1.docx]

Supplementary Material

1. **Supplemental Methods**
   1. **Aorta Plaque Quantification**

The aorta was isolated starting at the iliac bifurcation of the aorta, slowly moving up to the aortic arch. The aorta was cleared of excess outer adhesion and adventitial fat, placed in 5 mL of 10% neutral formalin and fixed overnight on a shaker at 4°C before being stained in Oil Red O. The staining process was performed as previously described (Maganto-Garcia et al., 2012). Briefly, aortas were washed overnight in PBS at 4°C, dehydrated for 2 min in propylene glycol and incubated for 2 hours at room temperature in 5 mL 0.5% Oil Red-O solution. The aortas were developed in a series of four dishes of 85% propylene glycol for 1 min each and washed overnight in 5 mL PBS at 4°C. The aortas were cut open to expose the inner face and atherosclerotic plaques. They were pinned on silicon-elastomers plates using stainless steel minutien pins and photographed for quantification. The number of atherosclerotic plaques in the ApoE-/- mice were counted and compared to control tissue from C57Bl/6J mice.

- 1. **Serum Cholesterol**

ApoE-/- mice naturally express higher levels of cholesterol (412 mg/dL) than C57BL/6 mice fed normal chow (127 mg/dL) (Yin et al., 2012). Total cholesterol (TC) was measured to ensure that a hypercholesteraemic state was achieved and that switching mice back to a normal chow did not affect the cholesterol levels (mice were switched over to a normal chow after surgery to potentially mimic a normal diet that a patient would adopt post-stroke). Serum was collected 2 days before stroke and a second time at euthanasia. The cholesterol assay was carried out with the Cholesterol Fluorometric Assay kit (Cayman Chemical No. 10007640) as per manufacturer’s instructions.

1. Su**pplemental Results**

The number of atherosclerotic plaques in the HFD-fed ApoE-/- - mice was significantly higher than that of control tissue (Suppl Fig 1A). Furthermore, mice in the ApoE-/- -HFD study had elevated levels of cholesterol levels at two time-points in the study; 2 days before stroke onset (saline: 1445 +/- 874.4 mg/dL, fingolimod: 1166 +/- 825.2 mg/dL), and one week after going off the HFD, at the time of euthanasia (saline: 491.7 +/- 52.42 mg/dL, fingolimod: 643.8 +/- 221.5 mg/dL) (Suppl Fig 1B). Both of these values are higher than average mouse values of ApoE-/- mice fed a standard chow (412 +/- 21 mg/dL) and similar to those of ApoE-/- on Cholesterol diet (629 mg/dL) (Yin et al., 2012).

1. **References**:

Maganto‐Garcia, E., Tarrio, M., Lichtman A.H. (2012). Mouse Models of Atherosclerosis. Curr. Protoc. Immunol. Chapter 15: 15.24.1-15.24.23. doi:10.1002/0471142735.im1524s96

Yin, W., Carballo-Jane, E., Mclaren, D. G., Mendoza, V. H., Gagen, K., Geoghagen, N. S., et al. (2012). Plasma lipid profiling across species for the identification of optimal animal models of human dyslipidemia. J. Lipid. Res. 53, 51-65. doi: 10.1194/jlr.M019927

## Supplementary Figures


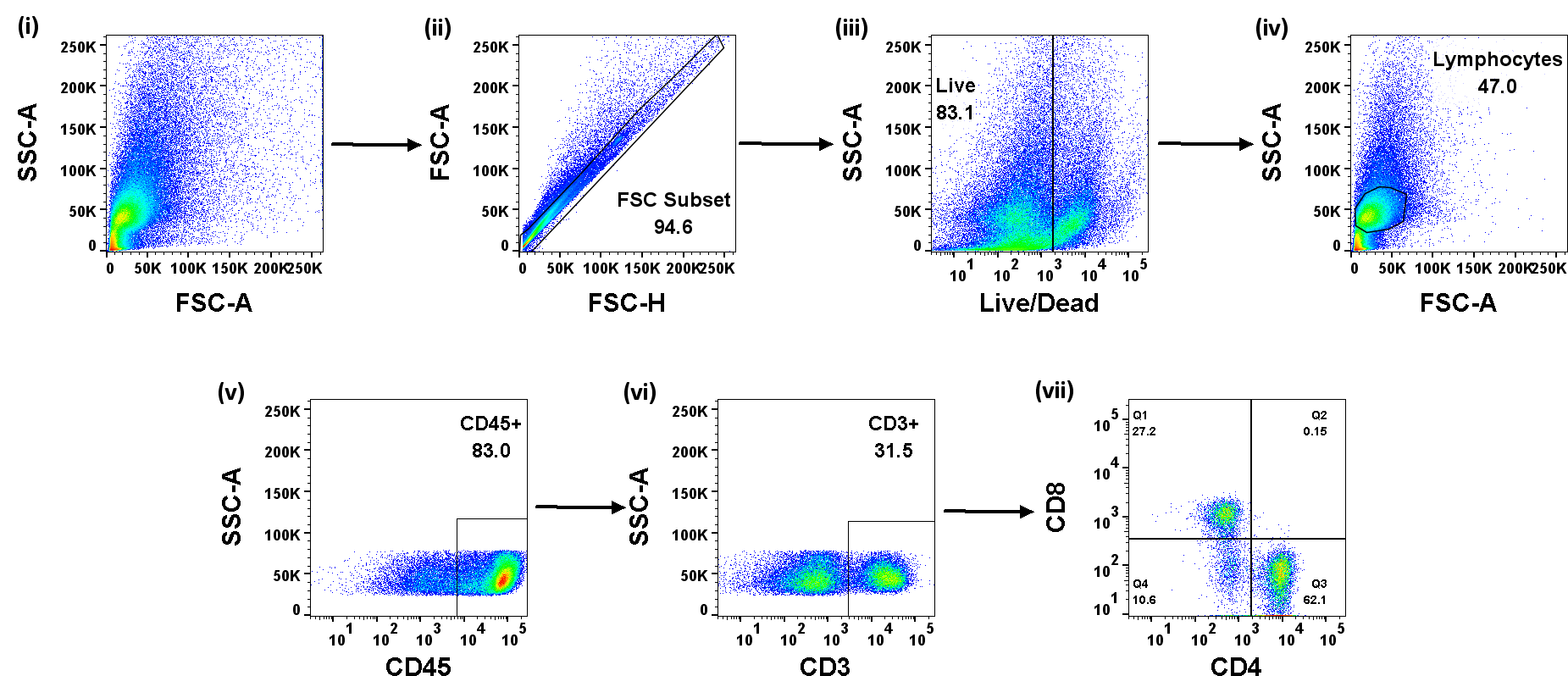


**Supplementary Figure 1:** Gating strategy for the determination of T cell subpopulations (i) = initial population, (ii) = singlets, (iii) = live cells, (iv) = lymphocytes, (v) = CD45+ cells, (vi) = CD3+ cells (T cells), (vii) = CD4+ T cells vs. CD8+ T cells.


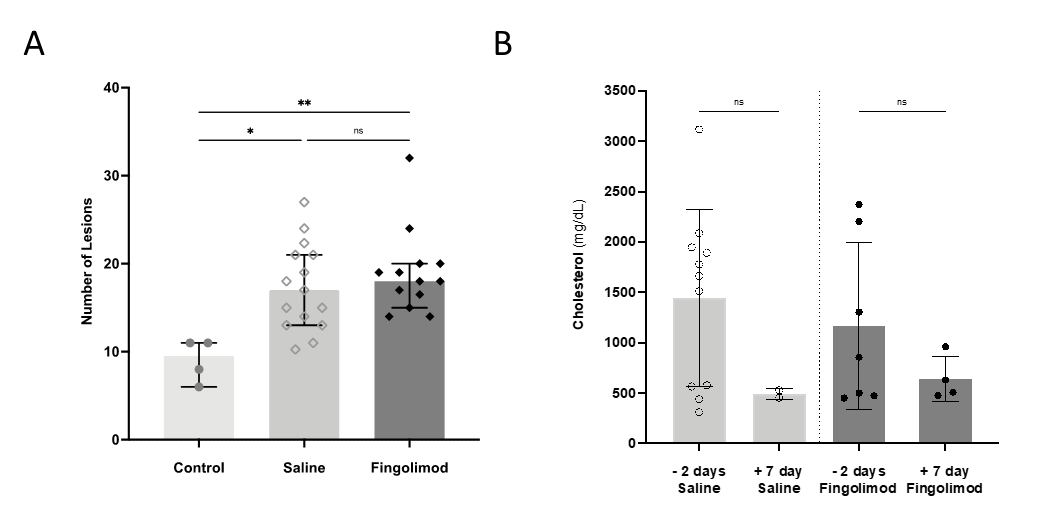


**Supplementary Figure 2:** A) Atherosclerotic plaque burden in control male C57BL/6JOlaHsd mice of 7-18 weeks of age and in mice and in HFD-fed ApoE-/- - mice in both treatment groups at the time of euthanasia 7 days after stroke onset. B) Serum cholesterol values measured 2 days before stroke and a second time at euthanasia.

**
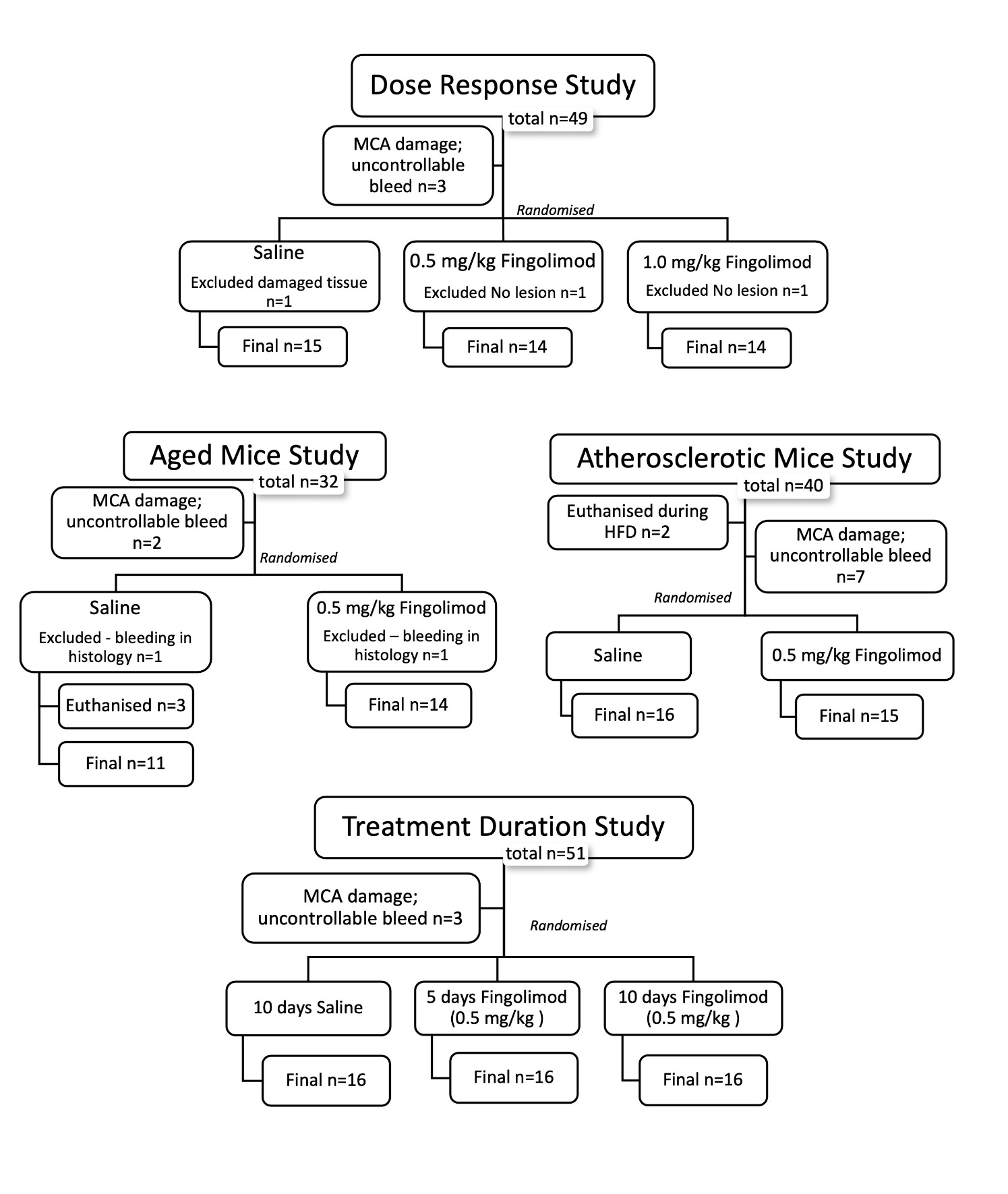
**

**Supplementary Figure 3:** Diagram illustrating the total number of mice that entered the different sub-studies, and the mice included and excluded from the final analysis.


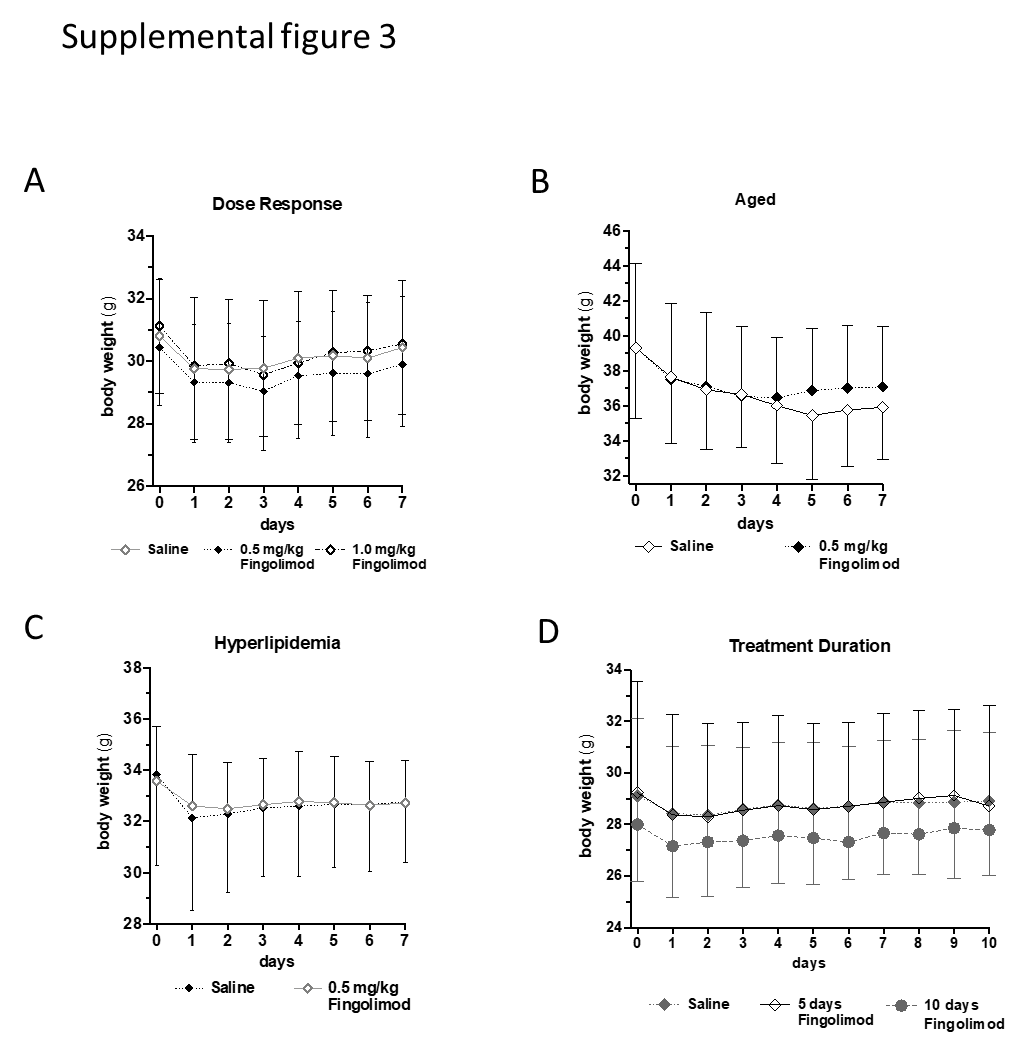


**Supplementary Figure 4:** Body weights of the mice used in the different sub-studies starting on the day of stroke onset until the day of euthanasia.


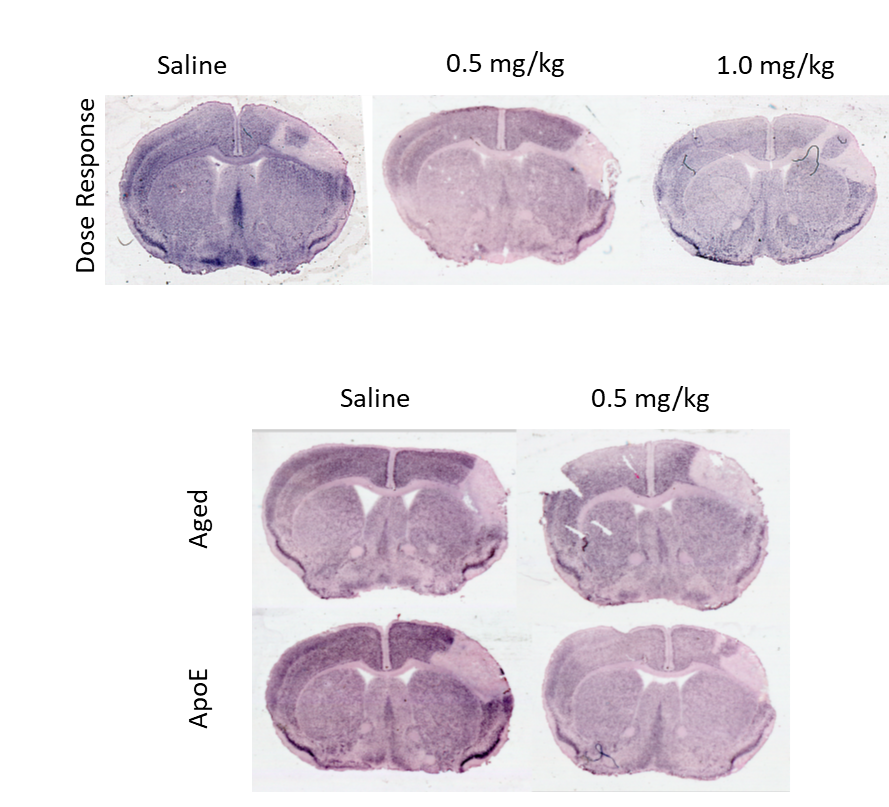


**Supplementary Figure 5:** Representative histological images of NeuN-stained sections (see main text for method) illustrating the size and location of the lesions (pale areas in the somatosensory cortex on the right side of the sections). Only one section is shown for each representative mouse brain, at the level of the anterior commissure, but infarct volumes were measured throughout the whole rostro-caudal extent of the lesion.

**
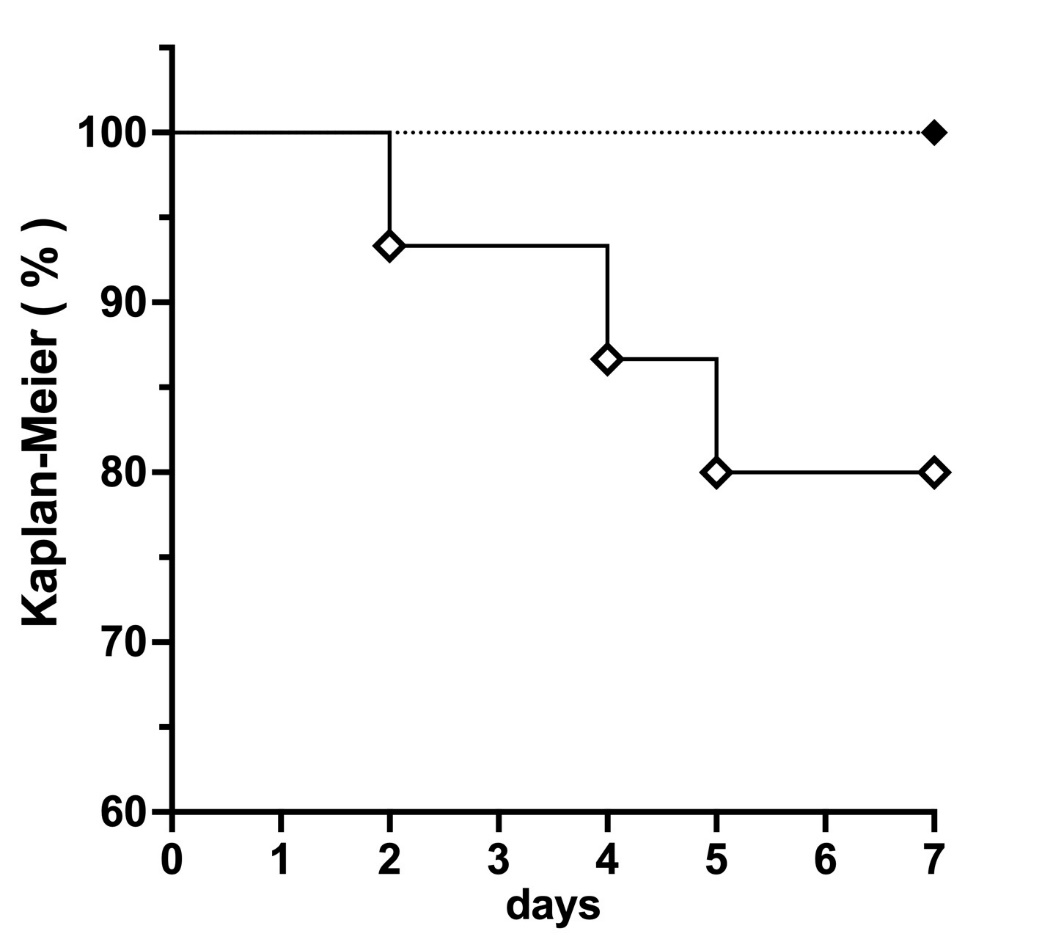
**

**Supplementary Figure 6**: Mortality in saline- and 0.5mg/kg fingolimod-treated aged mice. 3 mice, all in the saline group, had to be euthanised after reaching the threshold score for a humane endpoint at days 2, 4, and 5 post-stroke, respectively. A Log-rank test of the survival curve revealed that there was only a trend for improved survival in the 0.5mg/kg fingolimod treatment group (p=0.073).


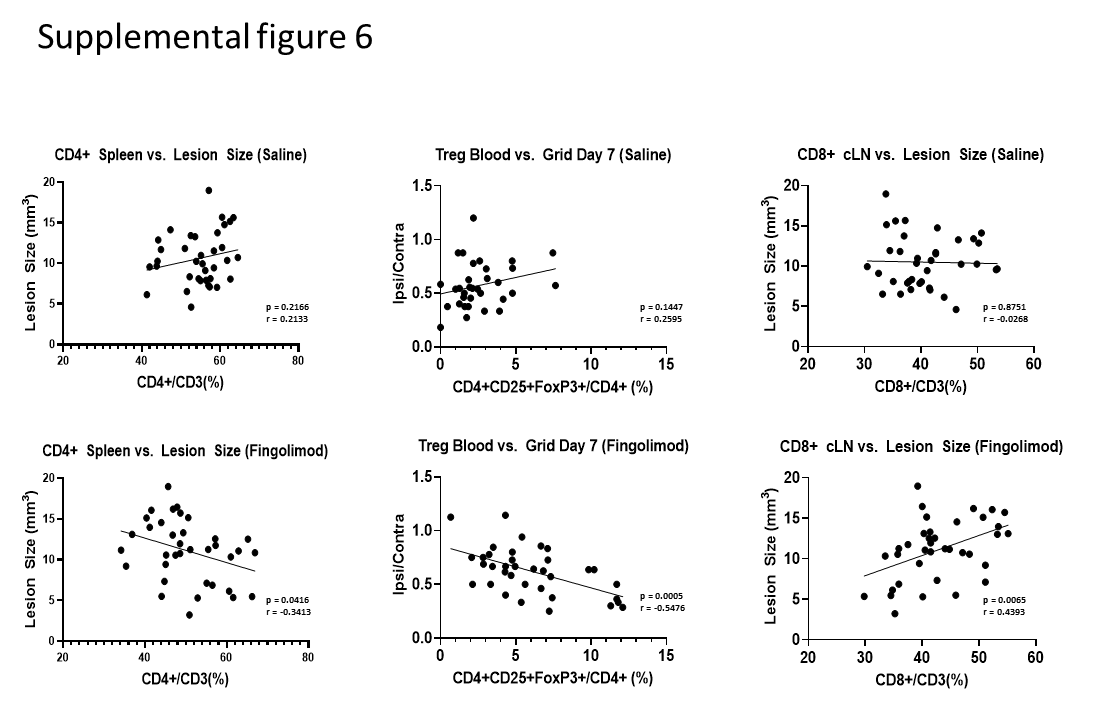


**Supplementary Figure 7:** Specific examples of correlations between outcome measures (lesion size and grid score at day 7) and flow cytometry data (CD4+ cell frequencies in spleen, Treg cell frequencies in blood, CD8+ cell frequencies in cervical, draining, lymph nodes). Pooled data from saline- and 0.5mg/kg fingolimod treated mice (n=37 per treatment group) were used to generate these graphs. All values were obtained 7 days after stroke onset.
